# BSA-based nanoassembly for combined chemo-photodynamic therapy of breast cancer

**DOI:** 10.1039/d6ra05429c

**Published:** 2026-09-25

**Authors:** Yunyang Zhao, Yingying Han, Yanni Zhang, Zaifeng Chen, Qiufang Gong, Jingbo Dong, Chao Liang

**Affiliations:** a Cixi Institute of Cancer Research, Cixi People's Hospital, Wenzhou Medical University Ningbo Zhejiang 315300 China liangchao@wmu.edu.cn; b Institute for Advanced Research, Cixi Biomedical Research Institute, Wenzhou Medical University Wenzhou Zhejiang 325000 China

## Abstract

The integration of photodynamic therapy and chemotherapy represents a promising strategy for precision cancer treatment, offering advantages including minimal invasiveness, reduced side effects, and enhanced therapeutic efficacy. In this study, we developed PTX/Ir@BSA nanoparticles through a self-assembly approach using bovine serum albumin (BSA) as a stabilizer to co-deliver the chemotherapeutic agent paclitaxel (PTX) and an iridium (Ir)-based photosensitizer. These nanoparticles demonstrated favorable stability and efficient reactive oxygen species (ROS) generation upon light irradiation. *In vitro* studies using 4T1 cells revealed that Ir@BSA generated substantial intracellular ROS under UV exposure, effectively inducing tumor cell death. When combined with PTX-mediated chemotherapy, the PTX/Ir@BSA nanoparticles exhibited remarkable antitumor effects. *In vivo* experiments further confirmed efficient tumor accumulation of the nanoparticles and significant tumor growth inhibition through this combined chemo-photodynamic approach. This work establishes a robust nanoplatform for developing combined chemo-photodynamic therapies, providing new opportunities for enhanced cancer treatment with reduced systemic toxicity and enhanced antitumor efficacy profiles.

## Abstract 2

PTX/Ir@BSA nanoparticles were fabricated through a self-assembly approach for PDT-mediated suppression of breast cancer growth.

## Graphical abstract

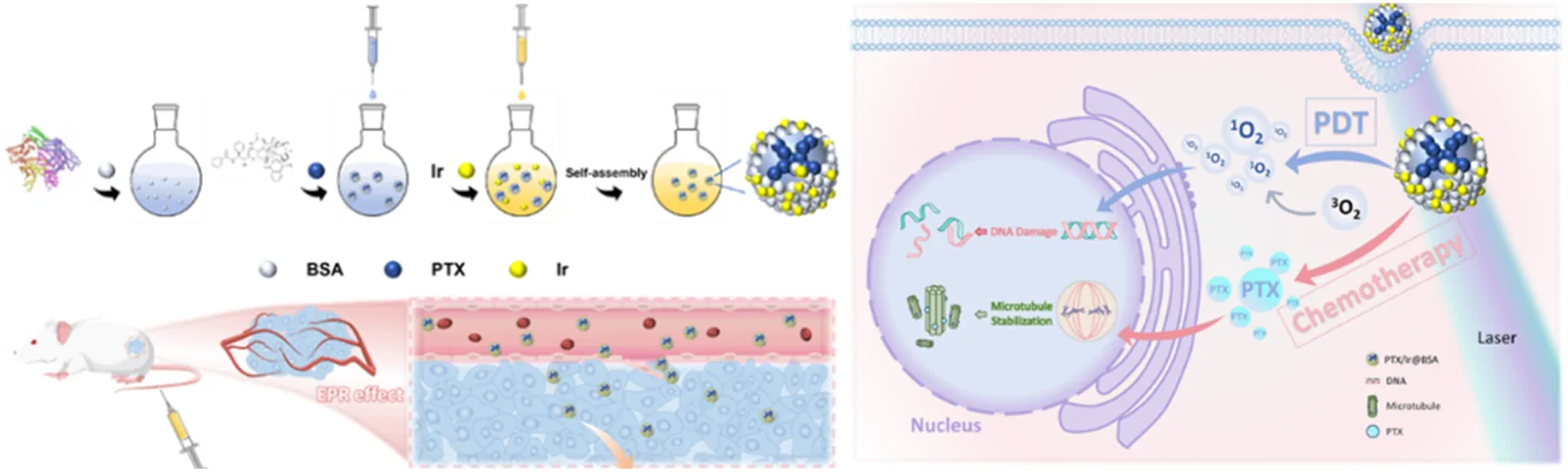

## Introduction

Cancer stands as a major global public health challenge, causing approximately 8.2 million deaths annually. Its high mortality rate and difficulty in treatment make it the foremost threat to human health.^1,2^ The three main conventional clinical treatment modalities each face limitations: surgical resection struggles to achieve complete tumor removal when boundaries between malignant and normal tissues are unclear, while perioperative complications significantly impact patients' quality of life.^3^ Although chemotherapy is widely used across cancer stages, tumor heterogeneity often leads to drug resistance, and its nonspecific cytotoxic mechanisms cause severe side effects such as myelosuppression and organ toxicity, substantially limiting clinical efficacy.^4–6^ This critical situation urgently demands the development of novel therapeutic strategies that maintain antitumor efficacy while reducing adverse effects.

In recent years, the establishment of multimodal combination therapy systems has brought breakthrough progress in cancer treatment.^7–9^ Among these, photodynamic therapy (PDT) has emerged with unique advantages: this technology utilizes tumor-selective photosensitizers that generate reactive oxygen species (ROS) under specific wavelength laser excitation to achieve precise malignant cell destruction.^10–13^ Its high selectivity, minimal damage to normal tissues, and repeatable treatment significantly outperform conventional therapies. However, current photosensitizers face two major technical challenges: poor formulation stability caused by hydrophobicity severely affects drug efficacy, and lack of tumor targeting may induce phototoxicity in normal tissues.^14–17^ To address these key scientific issues, researchers have innovatively integrated nanotechnology with PDT through engineered nanocarriers, not only resolving photosensitizer delivery challenges but also providing an ideal platform for chemo-PDT combination therapy.^18–21^

This study makes a promising pre-clinical strategy by employing human serum albumin (BSA) nanocarriers to simultaneously load the classic chemotherapeutic drug paclitaxel (PTX) and a novel iridium complex photosensitizer (Ir) through molecular self-assembly technology, successfully constructing a PTX/Ir@BSA nanotherapeutic system.^22^ This system demonstrates the following outstanding advantages: (1) maintains favorable stability in physiological environments, ensuring drug integrity in circulation; (2) efficiently generates singlet oxygen under irradiation, meeting clinical requirements for photodynamic conversion efficiency; (3) achieves an enhanced tumor accumulation through enhanced permeability and retention (EPR) effects. *In vitro* experiments confirm this nanosystem significantly enhances tumor cell sensitivity to treatment, while *in vivo* evaluations demonstrate both effective tumor growth suppression and substantially reduced systemic toxicity compared to conventional therapies. This research not only provides new insights for overcoming tumor drug resistance but also pioneers a novel nanotechnology-mediated chemo-photodynamic combination therapy paradigm.

## Results and discussions

We successfully synthesized PTX/Ir@BSA nanoparticles using bovine serum albumin as a stabilizer through a nano-self-assembly approach. To screen the optimal fabrication ratio, the effect of different molar ratios of BSA to PTX on the self-assembly behavior and particle size of PTX@BSA nanoparticles was systematically investigated. As showed in Fig. S1, the obtained particle size was almost identical to that of pure BSA at a BSA/PTX molar ratio of 1 : 1, indicating no successful formation of assembled nanoparticles. With the increase of PTX feeding proportion, the hydrodynamic size of PTX@BSA gradually increased. Specifically, the particle sizes were approximately 20 nm, 70 nm, 135 nm and 200 nm at BSA/PTX molar ratios of 1 : 5, 1 : 6, 1 : 10 and 1 : 20, respectively. Considering the optimal particle size range (10–200 nm) for tumor EPR effect and the requirement for subsequent efficient loading of iridium photosensitizer, the molar ratio of 1 : 10 was selected as the optimal formulation condition. Under this optimized ratio, the drug loading content and encapsulation efficiency of PTX were 0.77% and 84.3%, while those of the iridium complex were 5.7% and 60%, respectively, as shown in Tables S1, S2 and Fig. S2, S3. It has similar release profiles consistent with those previous work,^23,24^ achieving efficient co-encapsulation of chemotherapeutic and phototherapeutic agents within a single BSA nanoplatform. The morphology of the nanoparticles was characterized using a bio-transmission electron microscope (TEM) after negative staining with phosphotungstic acid. As shown in Fig. 1a, the synthesized nanoparticles exhibited a spherical structure with uniform distribution. The average particle size was determined by Litesizer Dynamic Light Scattering (DLS) analysis (Fig. 1b). Compared to free BSA, the assembled nanoparticles showed an increased size of approximately 150 nm, which was consistent with TEM observations, confirming successful nanoparticle synthesis. UV-vis spectroscopy in Fig. 1c revealed that PTX/Ir@BSA nanoparticles displayed the characteristic absorption peak of PTX at 230 nm, further demonstrating successful PTX loading. Fluorescence spectroscopy showed that compared to free Ir photosensitizer, both PTX/Ir@BSA nanoparticles and Ir@BSA nanoparticles exhibited a blue shift of the maximum fluorescence emission peak from 550 nm to 500 nm, likely induced by aggregation effects (Fig. 1d).

**Fig. 1:**
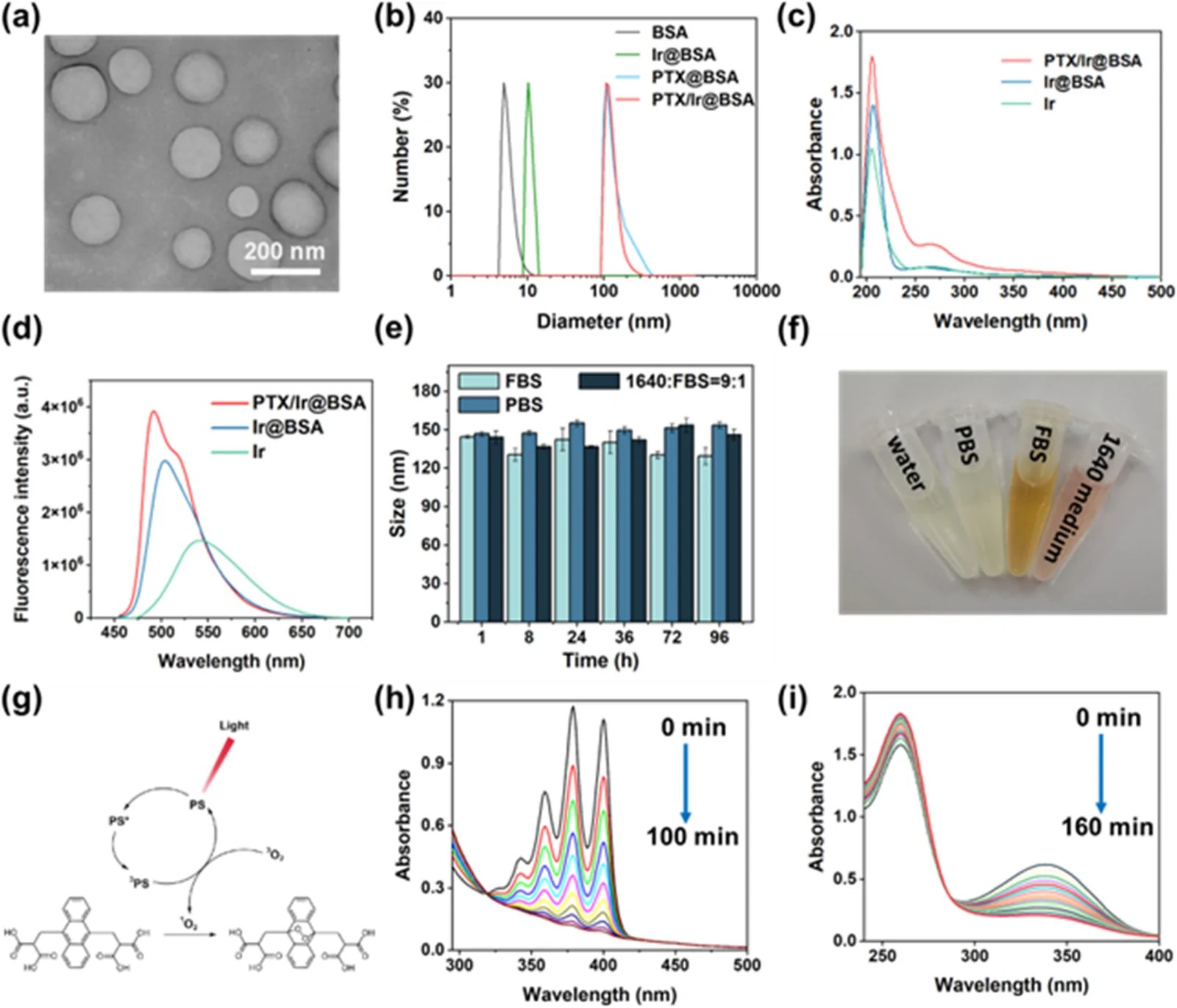
Characterization of the PTX/Ir@BSA nanoparticles. (a) Bio-transmission electron microscope image of PTX/Ir@BSA nanoparticles. (b) The diameter of PTX/Ir@BSA nanoparticles measured using DLS. (c) UV-vis spectra of Ir, Ir@BSA, and PTX/Ir@BSA. (d) The fluorescence spectrum of Ir, Ir@BSA, and PTX/Ir@BSA nanoparticles. (e) The diameter of PTX/Ir@BSA nanoparticles incubated in FBS, PBS and 1640 culture medium for different time points. (f) Photos of PTX/Ir@BSA nanoparticles incubated in FBS, PBS and 1640 culture medium. (g) Schematic illustration of the ABDA assay principle for singlet oxygen detection. (h) Absorption changes of ABDA in the presence of PTX/Ir@BSA nanoparticles under light irradiation. (i) Absorption changes of NADH in the presence of PTX/Ir@BSA nanoparticles under light irradiation.

Furthermore, we systematically evaluated the stability of the nanoparticles. When PTX/Ir@BSA nanoparticles were dispersed in various physiological solutions including FBS, PBS, and 1640 culture medium, dynamic light scattering measurements showed no significant changes in particle size after 96 hours of incubation, with no observable precipitation, indicating favorable stability under physiological conditions (Fig. 1e&f). Based on these results, we further investigated their photodynamic properties. For singlet oxygen generation assessment, 40 µL of 5 mM ABDA solution was mixed with 2 mL nanoparticle solution and exposed to UV irradiation. The decrease in ABDA absorbance at 378 nm was monitored *via* UV-vis spectrophotometry to quantify ^1^O_2_ production (Fig. 1g&h). However, negligible absorbance decay was observed in the control groups as showed in Fig. S4. NADH was used to monitor the generation of superoxide anion (O_2_˙^−^). The oxidation rate of NADH was measured by tracking absorbance changes at 339 nm for a solution containing 5 µM nanoparticles and 100 µM NADH under UV irradiation at different time intervals (Fig. 1i). The increase in the absorbance at 339 nm further demonstrated the generation of ROS under irradiation with no changes in the control groups in Fig. S5. To further directly verify the ^1^O_2_ generation capacity of PTX/Ir@BSA, electron spin resonance (ESR) measurements were carried out using TEMP as the specific spin-trapping reagent. Upon 395 nm LED irradiation, three equal-intensity characteristic peaks with a relative intensity ratio of 1 : 1 : 1 assigned to the TEMPO adduct were clearly recorded as showed in Fig. S6. This direct ESR evidence confirms that the PTX/Ir@BSA nanosystem can efficiently produce ^1^O_2_ under UV light irradiation, which is consistent with the indirect ROS detection results from ABDA and NADH assays. Taken together, the ESR characterization firmly demonstrates the favorable photodynamic performance of PTX/Ir@BSA and supports its photodynamic therapeutic mechanism.

The fabrication and self-assembly mechanism of the PTX/Ir@BSA combined chemo-photodynamic nanoplatform is illustrated in Scheme 1. BSA molecules possess abundant hydrophobic domains and numerous functional groups including amino, carboxyl, and hydroxyl groups in aqueous PBS solution. To reduce the system free energy, the hydrophobic moiety of PTX spontaneously binds to the hydrophobic cavities of BSA *via* hydrophobic interaction, preliminarily forming a stable self-assembled structure. Meanwhile, the hydroxyl groups of PTX can form hydrogen bonds with the polar functional groups of BSA, providing specific binding sites for the assembly of BSA and PTX. In addition, electrostatic interaction, van der Waals force, and π–π stacking further strengthen the intermolecular binding, synergistically driving the self-assembly process and maintaining the dynamic structural stability of PTX@BSA nanoparticles. For the modification of iridium complex, the trivalent iridium center endows the complex with specific charge distribution, thereby generating electrostatic attraction with the charged amino acid residues on the BSA surface. The hydrophobic phenylpyridine ligands of the iridium complex can be embedded into the hydrophobic inner cavities of BSA through hydrophobic interaction. Importantly, the empty orbitals of Ir(iii) can coordinate with lone-pair electron-containing atoms in BSA molecules, forming stable coordination bonds. Moreover, hydrogen bonding between the iridium complex and BSA further promotes assembly and structural stability. Driven by the above multiple synergistic interactions, the iridium complex is firmly immobilized on the surface of PTX@BSA, ultimately achieving the successful construction of uniform and stable PTX/Ir@BSA nanoparticles.

**Scheme 1:**
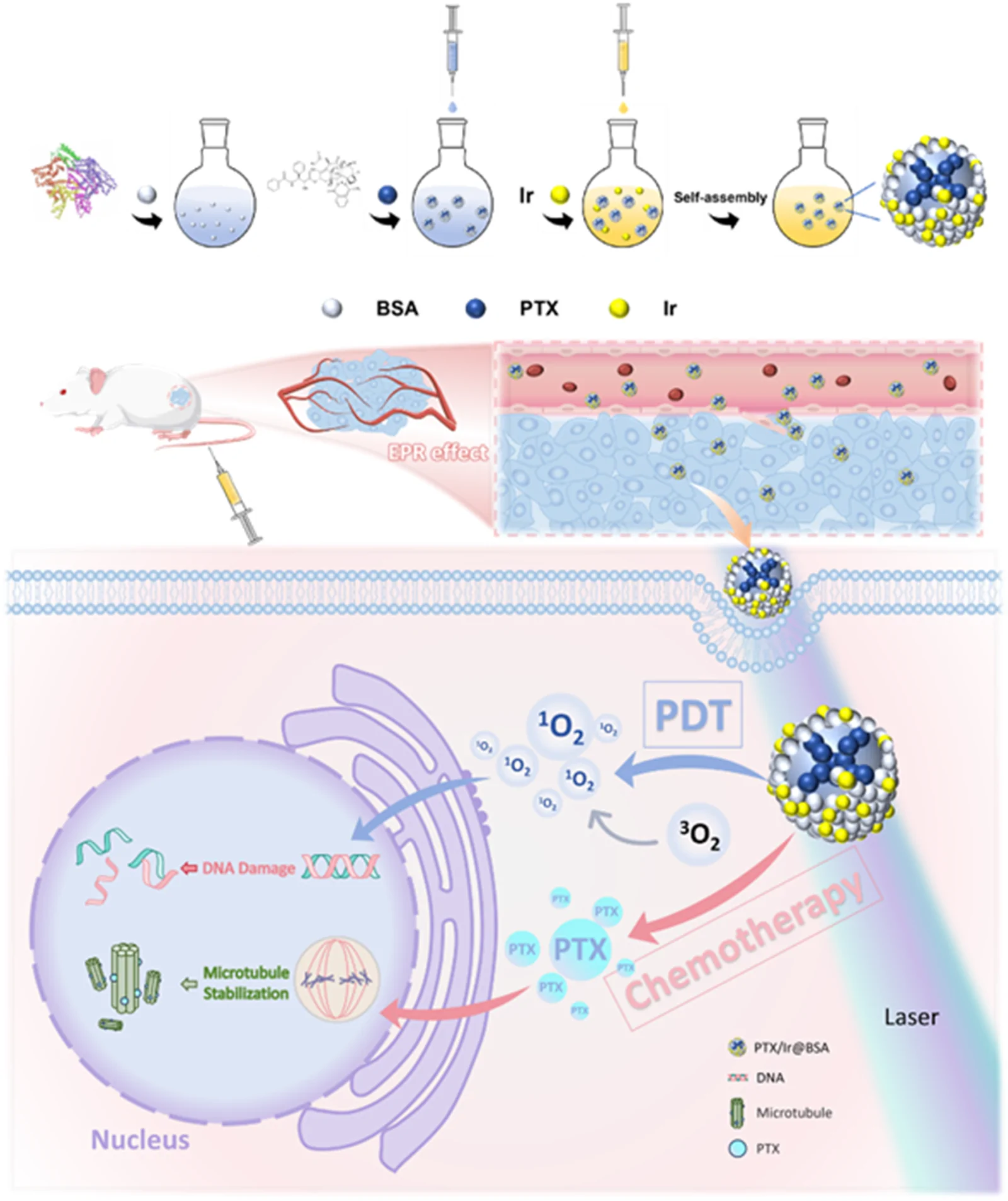
Illustration of PTX/Ir@BSA guided chemo-PDT combinational therapy.

To validate the cellular uptake of PTX/Ir@BSA nanoparticles by cancer cells, 4T1 cells were incubated with the nanoparticles at 37 °C for 0.5, 2, 4, 8, 10, and 12 hours, respectively, followed by Hoechst 33258 fluorescent staining to observe nanoparticle internalization. The results in Fig. 2a demonstrated a time-dependent increase in red fluorescence signals within cancer cells, indicating efficient cellular uptake of the nanomaterial. Building on these findings, we conducted cell-level therapeutic experiments. The MTT assay revealed that PTX@BSA exhibited stronger tumor cell-killing efficacy than free PTX, likely attributable to enhanced cellular uptake of the nanomaterial (Fig. 2b). We further evaluated the cytotoxicity of the nanoparticles with and without light irradiation. As shown in Fig. 2c, both PTX/Ir@BSA and Ir@BSA displayed low cytotoxicity in the absence of light. However, under light irradiation, Ir@BSA generated substantial singlet oxygen *via* photodynamic effects, significantly reducing tumor cell viability. When combined with PTX, PTX/Ir@BSA demonstrated the most potent cytotoxic effect, confirming that the combination therapy effectively suppresses tumor cell survival.

**Fig. 2:**
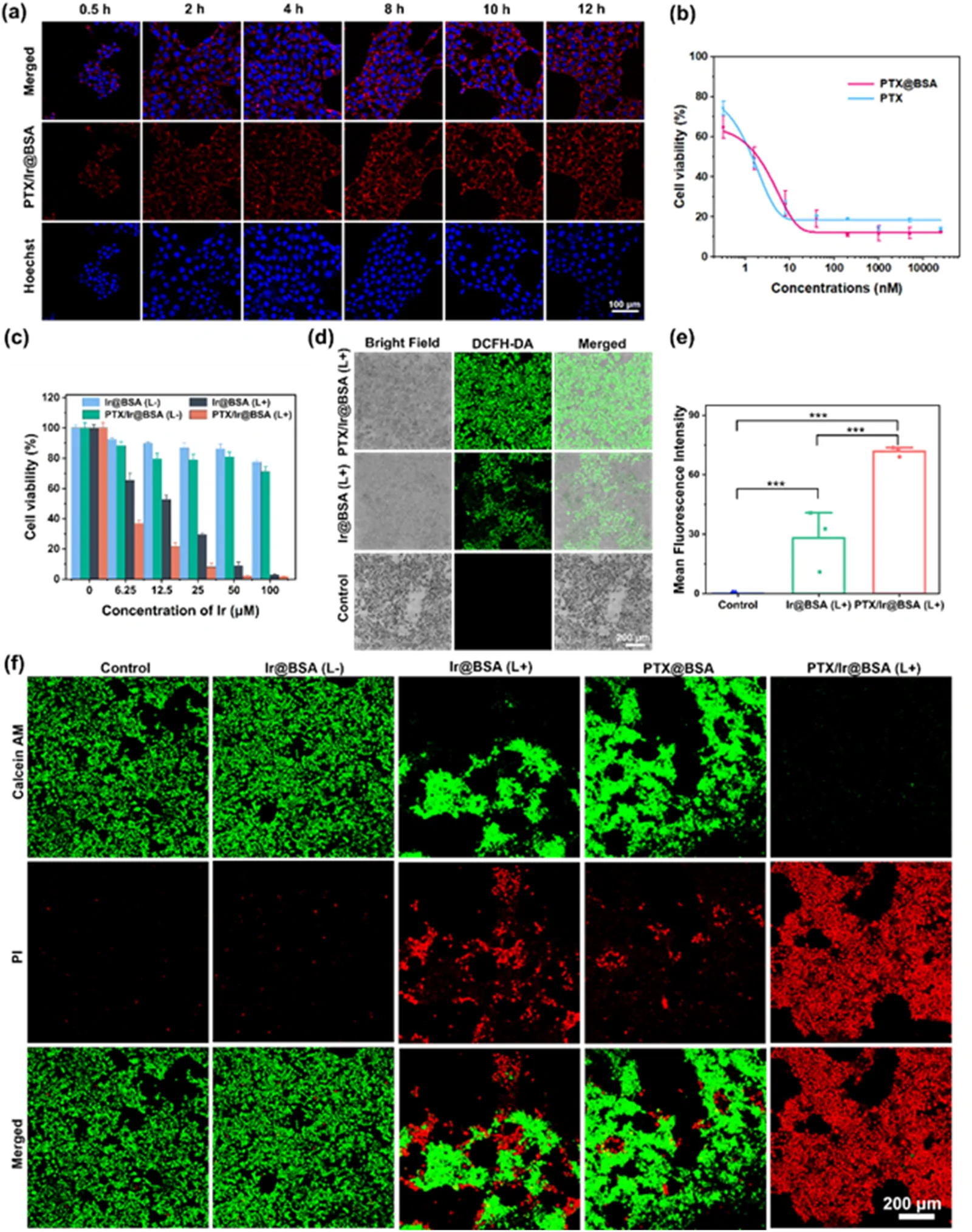
*In vitro* experiments. (a) CLSM images of cellular uptake of PTX/Ir@BSA at different time points. (b) Viability of 4T1 cells after incubation with various doses of PTX and PTX@BSA. (c) Viability of 4T1 cells after incubation with various nanoparticles with or without UV irradiation. (d) CLSM images of ROS in cells *via* DCFH-DA staining assays. (e) Quantification analysis of fluorescence intensity in (d). (f) Fluorescence microscope images of calcein-AM (green, live cells) and propidium iodide (red, dead cells) co-cultured 4T1 cells under different conditions.

Using the DCFH-DA probe, we further verified intracellular ROS generation under light irradiation, including hydroxyl radicals and peroxides. Fig. 2d&e showed that PTX/Ir@BSA-treated cells exhibited the strongest green fluorescence signal compared to Ir@BSA, indicating highly efficient ROS production. This enhanced effect may arise from PTX-induced mitochondrial damage, which potentiates photodynamic therapy to amplify ROS levels. Furthermore, the anticancer efficacy of PTX/Ir@BSA nanoparticles was assessed by live/dead cell staining (Fig. 2f). In the dark group, cytotoxicity increased with drug concentration, while light irradiation significantly enhanced the therapeutic outcome. These results were consistent with the MTT assay findings.

Targeting capability is crucial for enhancing the therapeutic efficacy of anticancer drugs. The real-time distribution and accumulation of Cy5.5-labeled nanoparticles were monitored using non-invasive intravital fluorescence imaging. Following intravenous administration, 4T1 tumor-bearing mice were imaged at 1, 2, 4, 8, 12, and 24 hours post-injection. Fluorescence imaging revealed gradual signal enhancement in tumor regions after PTX/Ir@BSA nanoparticle injection, peaking at 2 hours *via* the enhanced permeability and retention (EPR) effect (Fig. 3a&b). Meanwhile, blood circulation tests confirmed prolonged systemic circulation and efficient tumor accumulation of the nanoparticles (Fig. 3c). To study the biosafety of PTX/Ir@BSA nanoparticles, H&E-stained tissue sections (heart, liver, spleen, lungs, kidneys) collected on days 1 and 7 post-injection showed no significant pathological changes, demonstrating the minimal organ toxicity of PTX/Ir@BSA nanoparticles (Fig. 3d).

**Fig. 3:**
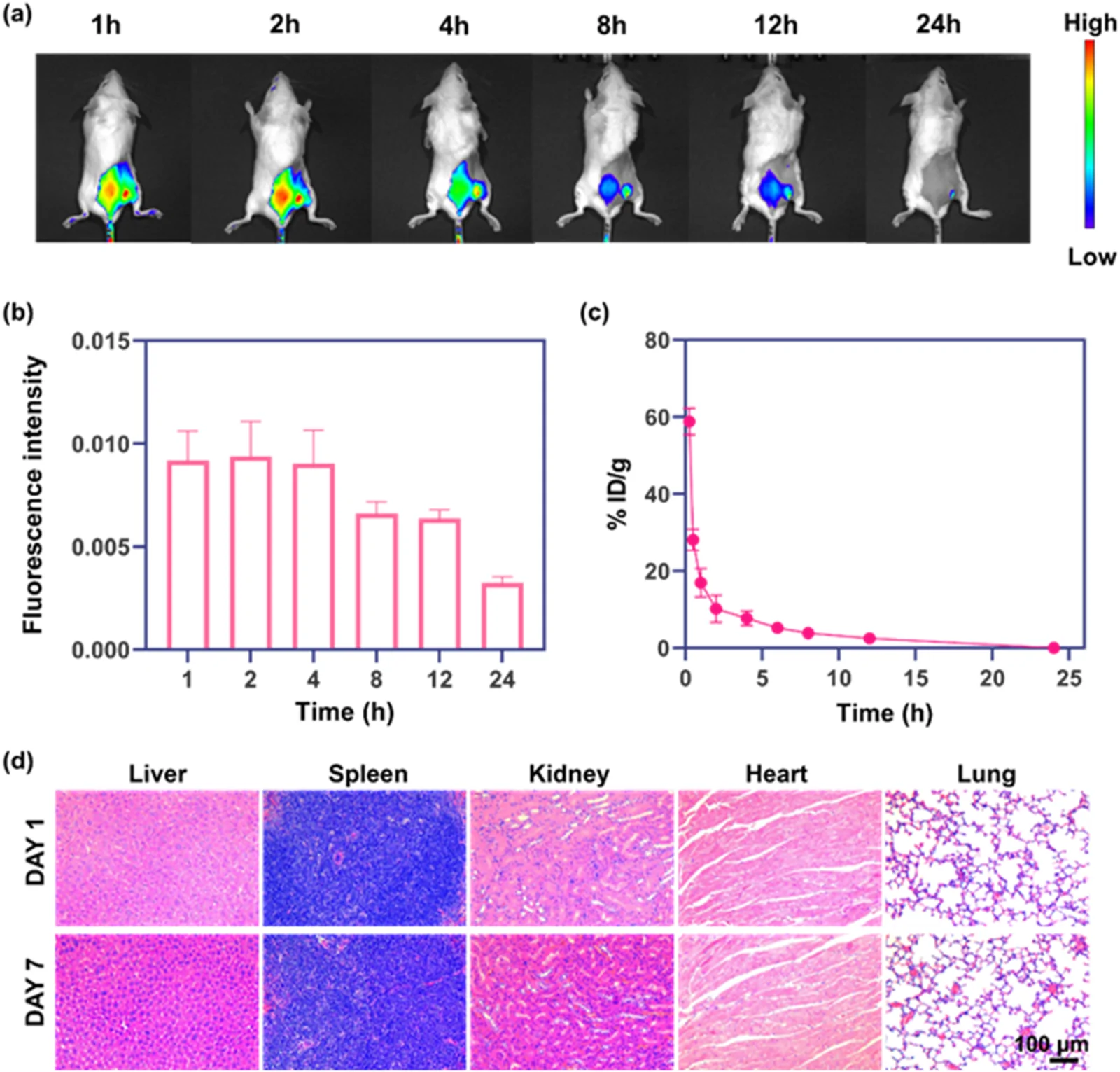
*In vivo* behavior of PTX/Ir@BSA nanoparticles. (a) *In vivo* fluorescence imaging of mice after i.v. of PTX/Cy5.5@BSA. (b) Quantification analysis of fluorescence intensity in (a). (c) Blood circulation of PTX/Cy5.5@BSA. (d) H&E staining slices of major organs (heart, liver, spleen, lungs, kidneys).

The anti-tumor potential of PTX/Ir@BSA nanoparticles was corroborated by using 4T1 tumor-bearing mice (Fig. 4a). The *in vivo* antitumor activity was further investigated by intravenously injecting PTX/Ir@BSA nanoparticles into 4T1 tumor-bearing mice. During the treatment period, the body weight and tumor volume of mice were monitored regularly. As evidenced by the tumor growth curves and tumor weight measurements (Fig. 4b–d), the saline control group exhibited rapid tumor progression within 14 days. While monotherapies (chemotherapy or photodynamic therapy alone) demonstrated certain tumor growth inhibitory effects, their therapeutic efficacy remained limited due to inherent treatment constraints. In contrast, the combination therapy group (chemotherapy-PDT) showed significantly enhanced antitumor efficacy with remarkable suppression of tumor growth. Histopathological examination (Fig. 4e) further revealed extensive tissue damage and cellular necrosis in tumors treated with PTX/Ir@BSA nanoparticles plus laser irradiation. These findings were consistent with the tumor growth curve data, collectively demonstrating that the combined application of PTX/Ir@BSA with light irradiation substantially potentiated the antitumor effects. Throughout the experimental period, all treatment groups maintained steady body weight gain (Fig. 4f), indicating the absence of significant systemic toxicity from the administered formulations.

**Fig. 4:**
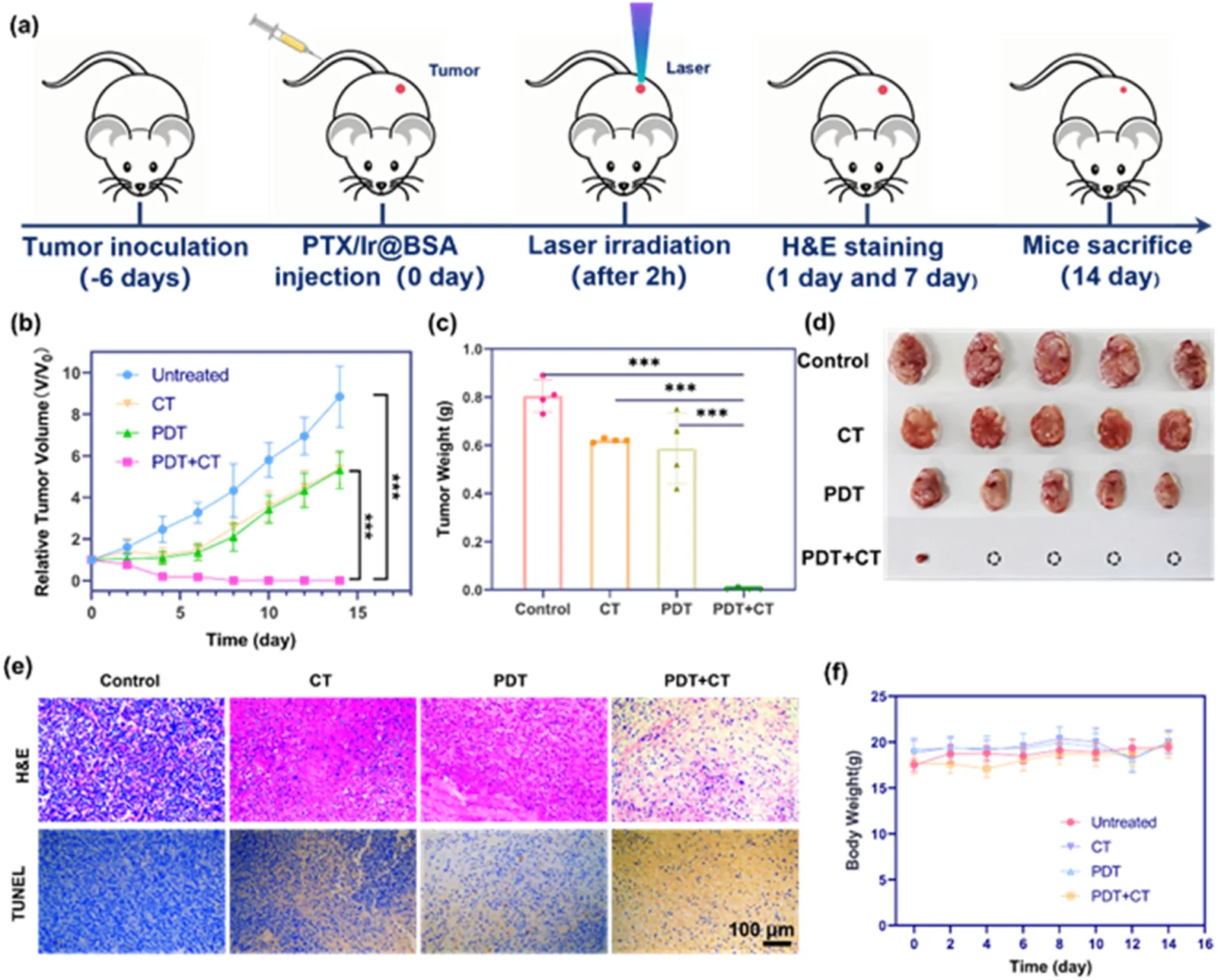
*In vivo* anti-tumor therapy. (a) A schematic illustration shows the therapeutic process on a mouse model. Tumor volume changes (b), tumor weight (c), and tumor images (d) of 4T1 bearing BALB/c mice after different treatment. (e) H&E and TUNEL staining assays after different treatment. (f) The body weight changes during therapy.

Compared with previously reported conventional BSA-based chemo-photodynamic co-delivery systems, most of which rely on simple physical encapsulation of organic photosensitizers and chemotherapeutic drugs, the PTX/Ir@BSA nanoplatform constructed in this work exhibits distinct structural and functional advantages.^25,26^ Different from physically assembled albumin nanoparticles that are prone to drug leakage and structural dissociation in physiological environments, our system introduces Ir(iii) complexes with coordination binding capability. The iridium photosensitizer is stably anchored on the BSA skeleton through coordination interaction, hydrophobic embedding and hydrogen bonding, which effectively improves the structural stability of nanoparticles and avoids premature burst release of therapeutic drugs. Meanwhile, different from free iridium complexes with severe aggregation-caused quenching and poor photodynamic performance in aqueous solution, BSA confinement can significantly optimize the dispersion state of iridium complexes and enhance singlet oxygen generation efficiency, thereby achieving superior chemo-photodynamic synergistic antitumor effects. Combined with the good biocompatibility and tumor enrichment ability of natural albumin, this nanoplatform realizes efficient co-delivery and synergistic therapy of chemotherapy and photodynamic agents, showing reliable antitumor efficacy both *in vitro* and *in vivo*.

Nevertheless, the current nanoplatform still has two inherent limitations that need to be further optimized in future research. First, consistent with most reported organoiridium-based PDT systems, the photodynamic activation of PTX/Ir@BSA depends on 395 nm near-ultraviolet light excitation. Limited by the shallow tissue penetration and potential normal tissue phototoxicity of UV light, this therapeutic strategy is more suitable for superficial and local tumor treatment, while its application in deep tumor therapy and large-area tumor treatment is restricted.^27^ Second, this work only focuses on the *in vitro* and *in vivo* therapeutic efficacy of the nanoplatform, and the long-term metabolic behavior, *in vivo* circulation stability and immune response of PTX/Ir@BSA still need further in-depth exploration.

In general, PTX/Ir@BSA nanodelivery system provides a feasible and high-efficiency strategy for chemo-photodynamic combined tumor therapy.^22^ Despite the above-mentioned limitations in excitation conditions and systematic biosafety evaluation, this work verifies the superiority of coordination-stabilized albumin nanocarriers over traditional physically loaded systems, and offers new insights for the design and construction of high-stability metal complex-based phototherapeutic nanoplatforms. Future research will focus on developing long-wavelength excitable iridium derivatives, introducing upconversion modification strategies, and supplementing comprehensive long-term biosafety detection, so as to further promote the clinical translational potential of such albumin-based synergistic therapeutic nanomedicines.

## Conclusion

In summary, PTX/Ir@BSA nanoparticles were successfully fabricated *via* a facile self-assembly strategy through the incorporation of iridium complexes and PTX for combined chemo-photodynamic therapy. This constructed nanosystem exhibits remarkable structural stability, which effectively alleviates premature drug leakage and guarantees efficient co-delivery of dual therapeutic agents to tumor lesions. Upon localized light irradiation at the tumor region, the encapsulated iridium complexes can effectively generate singlet oxygen for photodynamic therapy, while the released PTX exerts potent chemotherapeutic effects. Consistent with the combined therapeutic design, systematic *in vitro* and *in vivo* investigations demonstrate that PTX/Ir@BSA possesses outstanding combined antitumor performance. Although the current PDT strategy relies on 395 nm near-ultraviolet excitation with inherent limitations in tissue penetration and clinical translational adaptability, this BSA-based co-delivery nanoplatform still offers a reliable and promising strategy for the design and development of high-efficiency chemo-photodynamic therapeutic agents.

## Methods

### Preparation of nanoparticles

PTX/Ir@BSA: 20 mg PTX powder was dissolved in 1 mL methanol to prepare 20 mg mL^−1^ PTX stock solution (stored at 4 °C in dark). 2 mg iridium complex was dissolved in 50 µL DMSO. 20 mg BSA was dissolved in 10 mL PBS, and the solution was heated to 84 °C. Then 128 µL PTX stock solution was added dropwise and incubated at 84 °C for 10 min. Afterwards, 50 µL DMSO-dissolved iridium complex was added into the pre-formed PTX-loaded BSA solution, and the mixture was stirred at room temperature for 1 h. For purification, 15 kDa ultrafiltration tubes were used at 4 °C, 4000 rpm for 20 min per cycle; the ultrafiltration step was repeated twice to remove free PTX, DMSO and unloaded iridium complex. The retained retentate was re-suspended with PBS to obtain PTX/Ir@BSA.

Ir@BSA: 10 mg BSA was dissolved in ultrapure water, and absolute ethanol was dropwise added to form a white turbid solution. 10 µL glutaraldehyde was diluted to 200 µL with ultrapure water, then 10 µL diluted glutaraldehyde solution was added, followed by continuous stirring for 6 h. After the same ultrafiltration purification process, iridium complex was introduced to prepare Ir@BSA.

PTX@BSA was prepared following the identical procedure of PTX/Ir@BSA, except that iridium photosensitizer was omitted during fabrication.

The iridium complex [Ir(ppy)_2_(CH_3_CN)_2_] was modified and synthesized according to previously reported work from our collaborators.^28^ The brief synthetic procedure is as follows: phenylpyridine, IrCl_3_·3H_2_O, 2-ethoxyethanol and water were added into a 100 mL three-necked flask. The mixture was reacted at 110 °C under N_2_ atmosphere for 24 h under dark conditions. After cooling to room temperature, the crude product was collected by vacuum filtration and washed with ethanol to obtain a yellow solid. Subsequently, silver nitrate and acetonitrile were added, and the mixture was refluxed under N_2_ atmosphere for another 24 h. After cooling, vacuum filtration was performed to collect the concentrated filtrate. A small amount of dichloromethane was added to remove excess silver nitrate *via* vacuum filtration. The target iridium complex was finally obtained after concentrating the filtrate.

#### Characterization of nanoparticles

The average size of nanoparticles was determined by Litesizer DLS, and the morphology of the nanoparticles was examined using a bio-transmission electron microscope following negative staining with phosphotungstic acid.

#### Stability of PTX/Ir@BSA

Nanoparticles were individually dispersed in PBS, FBS, and 1640: FBS (9: 1) solutions, and the particle size was measured and documented at multiple time points within 96 hours.

#### Photodynamic effect of PTX/Ir@BSA

40 µL of 5 mM ABDA solution was combined with 2 mL nanoparticle solution and controlled to achieve an absorbance value of 2.0 at 378 nm. Subsequently, the photosensitizer-loaded nanoparticles were added to adjust the absorbance of the mixed solution to approximately 1.1 at 378 nm. The mixture was irradiated using a 395 nm LED light with power density of 0.4 W cm^−2^ for 5 min at each time interval, and the UV-vis absorption spectrum was recorded after each irradiation. The decrease in the characteristic absorption peak of ABDA at 378 nm was monitored to evaluate the ^1^O_2_ generation capacity of the nanoparticles. PBS solution containing 5 µM nanoparticles and 100 µM NADH was irradiated using a 395 nm LED light with power density of 0.4 W cm^−2^ for 5 min at each time interval, and the UV-vis absorption spectrum was also recorded after each irradiation. The alteration in absorbance of NADH at 339 nm was measured to quantify the oxidation rate of NADH for monitoring the generation of O_2_˙^−^.

#### Cell experiments

The 4T1 cells were incubated with the nanoparticles at 37 °C for 0.5, 2, 4, 8, 10, and 12 hours respectively, followed by staining with Hoechst 33258 fluorescent dye to investigate nanoparticle uptake by the cells.

4T1 cells were seeded in glass-bottomed dishes and cultured at 37 °C with a 5% concentration of CO_2_ for 24 hours. Then, PTX/Ir@BSA nanoparticles were incubated with the cells for 5 hours. Subsequently, the culture medium and materials were washed off with PBS, and DCFH-DA was incubated with the cells for 30 minutes. The cells were then exposed to UV irradiation for 5 minutes, and the generation of singlet oxygen in the cells was observed using a confocal microscope.

4T1 cells were implanted in 96-well plates and incubated with nanoparticles of varying concentrations for 72 hours. Cell viability was assessed using the MTT assay. Alternatively, 4T1 cells were seeded into glass-bottomed culture dishes and cultured at 37 °C in a 5% CO_2_ atmosphere for 24 hours. Subsequently, the cells were exposed to drug-loaded nanoparticles in a 1640 culture medium containing 10% FBS for 4 hours. Replace the culture medium with fresh medium, and expose the cells to UV irradiation for 5 minutes. Prepare a working solution of calcein AM and PI at the appropriate concentration, and incubate it with the cells in the confocal dish for 20 minutes. Live and dead cell staining images were acquired using a laser confocal microscope.

#### Tumor model

Female BALB/c mice were purchased from Beijing Vital River Laboratory Animal Technology Co. Ltd. Mice are housed in a SPF animal research facility. 25 µL RPMI 1640 culture medium containing one million 4T1 cells were subcutaneously injected into the right dorsal region of mice. Six days post-injection, mice harboring tumors measuring 150 mm^3^ in volume were selected for the experiment. The formula for calculating volume is expressed as follows: volume = 0.5 × length × width^2^. All animal procedures were performed in accordance with the Guidelines for Care and Use of Laboratory Animals of Wenzhou Medical University and approved by the Animal Ethics Committee of Wenzhou Medical University (SYXK 2021-0020). BALB/c female white mice, 6–8 weeks old (18–23 g), were purchased from Hunan SJA Laboratory Animal Co., Ltd, China and maintained under a 12 h light/dark cycle with free access to water and food.

#### *In vivo* anti-tumor therapy

The 4T1 tumor-bearing female BALB/c mice were randomly divided into 4 groups. (a) Control, (b) CT, (c) PDT, (d) PDT+CT. The mice in the (a) and (b) group were intravenously injected with 0.1 mL of PBS without and with PTX@BSA nanoparticles, while (c) and (d) groups of mice were injected intravenously with 0.1 mL of PBS containing 10 µM (based on the concentration of the iridium complex) Ir@BSA and PTX/Ir@BSA nanoparticles, respectively. At 2 h after injection, the tumor site was irradiated with a 395 nm LED lamp for 1 h at a power density of approximately 0.4 W cm^−2^. The tumor volume and mouse weight were recorded. The mice were sacrificed 14 days after treatment and their major organs and tumors were excised, imaged, and subsequently subjected to H&E staining and TUNEL staining for the assessment of pathological changes and tumor tissue apoptosis. Mice were monitored routinely throughout the treatment period. The humane endpoint was defined as tumour volume exceeding 1000 mm^3^, at which point the animals were humanely sacrificed.

#### *In vivo* behavior of PTX/Ir@BSA

Cy5.5 dye was loaded onto nanoparticles to formulate PTX/Cy5.5@BSA. Subsequently, three subcutaneous tumor-bearing mice were administered 200 µL of PTX/Cy5.5@BSA *via* tail vein injection, with a dosage of 4 mg mL^−1^ calculated based on the BSA content. Fluorescence imaging was performed on mice at 1, 2, 4, 8, 12, and 24 hours utilizing the IVIS Lumina.

#### Biological safety assessments

Five healthy female BALB/c mice were randomly selected and administered 500 µL of PTX/Ir@BSA *via* the tail vein at a dosage of 80 mg kg^−1^, based on the weight of BSA. The mice remained in good health, with no abnormalities observed 48 hours post-injection. Furthermore, two healthy female BALB/c mice were randomly selected and administered 200 µL of PTX/Ir@BSA *via* the tail vein, with the dosage being twice that of the therapeutic level. The major organs of the mice were subsequently stained with hematoxylin and eosin (H&E) on both day one and day seven.

## Conflicts of interest

There are no conflicts to declare.

## Supplementary Material

RA-016-D6RA05429C-s001

## Data Availability

The all data generated in this study have been deposited in the Zenodo. Other data related to this work are available from the corresponding authors upon reasonable request. Supplementary information (SI) is available. See DOI: https://doi.org/10.1039/d6ra05429c.
