# Supplementary material for: BSA-based nanoassembly for combined chemo-photodynamic therapy of breast cancer
Source: RSC Adv. 2026 Sep 25. Online ahead of print. doi: 10.1039/d6ra05429c (PMC13614019; doi:10.1039/d6ra05429c)
Supplement: Supplementary file 1 [file RA-OLF-D6RA05429C-s001.pdf]

Supplementary Information (SI) for RSC Advances.
This journal is © The Royal Society of Chemistry 2026
Supplementary Information (SI) for RSC Advances.
This journal is © The Royal Society of Chemistry

Supporting information for

BSA-based nanoassembly for Combined Chemo-Photodynamic Therapy of Breast Cancer

Yunyang Zhao,[‡,a] Yingying Han,[‡,b] Yanni Zhang,[a] Zaifeng Chen,[a] Qiufang Gong,[*,a] Jingbo Dong,[*,a] Chao Liang[*,a,b]

[a]Cixi Institute of Cancer Research, Cixi People's Hospital, Wenzhou Medical University, Ningbo, Zhejiang, 315300, China
[b]Institute for Advanced Research, Cixi Biomedical Research Institute, Wenzhou Medical University, Wenzhou, Zhejiang, 325000, China

‡ These authors contributed equally.

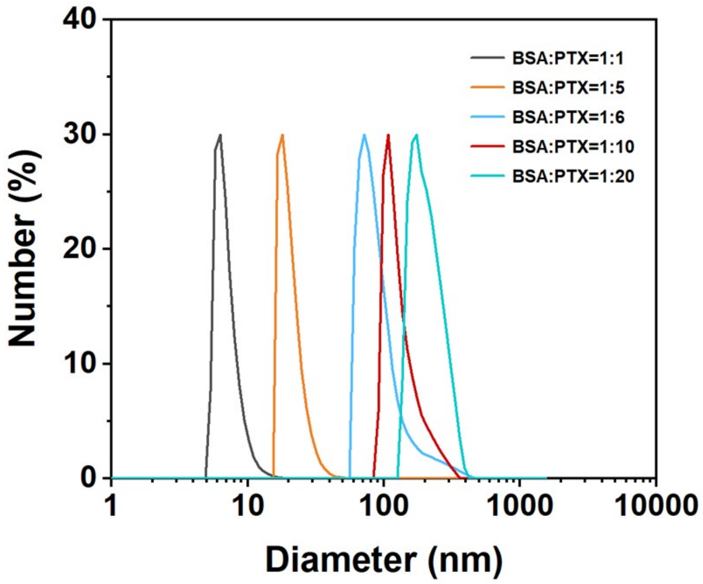

Figure S1. Hydrodynamic sizes of PTX@BSA nanoparticles measured by Dynamic Light Scattering (DLS) at different BSA/PTX feeding molar ratios.

Table S1. Encapsulation efficiency and drug loading efficiency of paclitaxel (PTX).

PTX in PTX/Ir@BSA	Concentration, μg/mL	Mass, μg	Encapsulation efficiency, %	Loading efficiency, %
Encapsulated drug	30.85	154.27	84.25	0.77
Free drug	3.20	28.84		

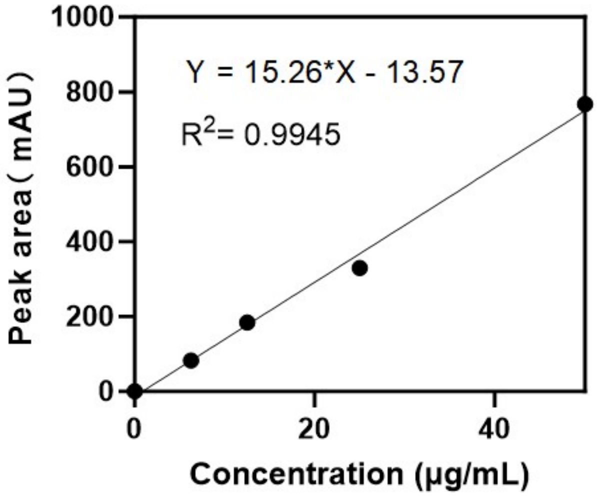

Figure S2. HPLC standard calibration curve for PTX.

Table S2. Encapsulation efficiency and drug loading efficiency of Ir complex.

Ir complex in PTX/Ir@BSA	
Absorbance	1.895
Concentration, μg/mL	1396.893
Mass, μg	1198.534
Encapsulation efficiencies, %	59.93
Loading efficiency, %	5.65

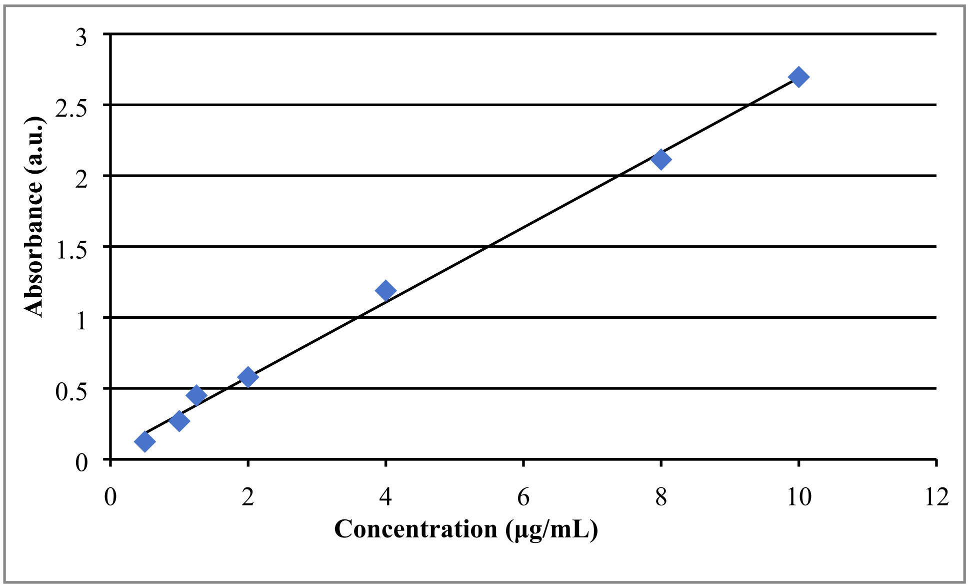

Figure S3. Standard calibration curve of the Ir complex measured by UV-vis spectroscopy.

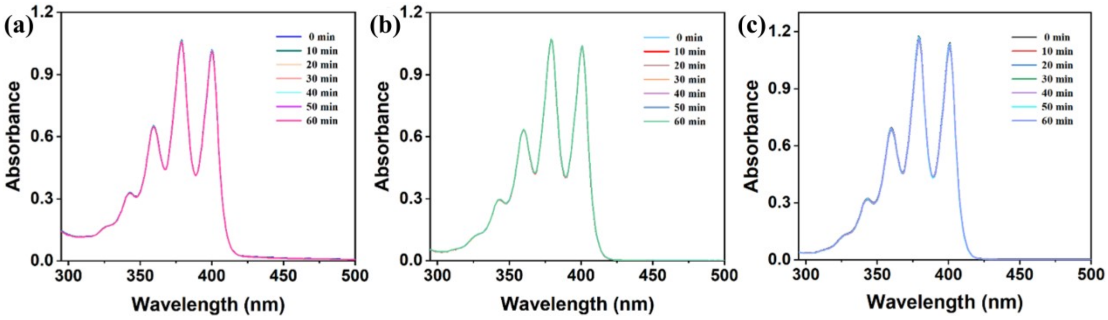

Figure S4. UV-Vis absorption spectra of the mixed PTX/Ir@BSA and ABDA solution under static conditions (a), ABDA upon UV irradiation (b), and the mixed Ir@BSA and ABDA solution under static conditions (c).

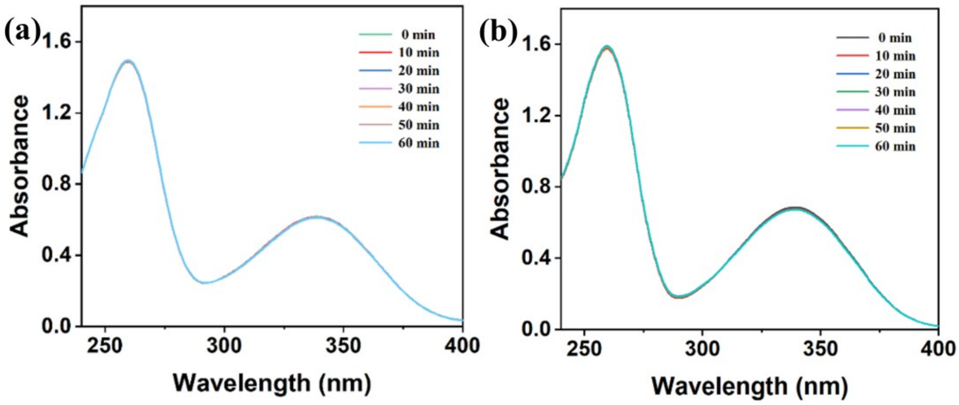

Figure S5. UV-Vis absorption spectra of the mixed PTX/Ir@BSA and NADH solution under static conditions (a) and NADH upon UV irradiation (b).

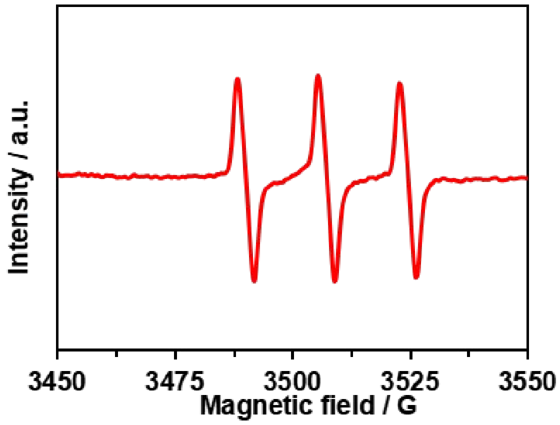

Figure S6. ESR spectra of TMP-$^1O_2$ adducts for PTX/Ir@BSA nanoparticles with 395 nm light irradiation.
